# Supramolecular Polymers With AIE Property Fabricated From a Cyanostilbene Motif-Derived Ditopic Benzo-21-Crown-7 and a Ditopic Dialkylammonium Salt

**DOI:** 10.3389/fchem.2020.610093

**Published:** 2020-11-19

**Authors:** Haoran Wu, Tangxin Xiao

**Affiliations:** School of Petrochemical Engineering, Changzhou University, Changzhou, China

**Keywords:** supramolecular polymer, AIE, host-guest, fluorescent materials, B21C7

## Abstract

Fluorescent supramolecular polymers (FSP) have attracted considerable attention in recent years. Particularly, the incorporation of aggregation-induced emission (AIE) property to the FSP will bring this material into practical applications. Herein, we designed and synthesized a cyanostilbene motif derived ditopic benzo-21-crown-7 (B21C7) as a host molecule (**H**). The cyanostilbene motif endows **H** with AIE property while the B21C7 motif renders it with the capability to complex with electron deficient guest molecules. Upon the addition of a ditopic dialkylammonium salt molecule (**G**), a novel FSP with blue luminescent property can be constructed. This B21C7-based host-guest FSP with blue fluorescence may have potential application in supramolecular luminescent materials.

## Introduction

Supramolecular polymers, in which ordered and highly directional polymeric arrays of monomeric building blocks are brought together by reversible non-covalent bonds, are outstanding materials that generally exhibit stimuli-responsive and self-healing properties (Aida et al., 2012; Yang et al., 2015; Wehner and Würthner, 2020). The driving force of supramolecular polymers usually includes hydrogen bonds (Xiao et al., 2019b,e; Datta et al., 2020; Qi et al., 2020), metal-ligand bonds (Zheng et al., 2016; Shi et al., 2019), host-guest complexation (Guo et al., 2010; Harada et al., 2014; Wang et al., 2018; Xiao and Wang, 2018; Xiao et al., 2018, 2019d; Chen et al., 2019), donor–acceptor interaction (Han et al., 2018), π-π stacking (Wagner et al., 2019; Xiao et al., 2019c), or a combination of them (Li et al., 2012; Wei et al., 2015; Xiao et al., 2020b). In recent years, supramolecular fluorescent materials have drawn much attention, such as fluorescent molecular switches (Cheng et al., 2017), fluorescent sensors (Kumawat et al., 2019), fluorescent metallaclip (Wang et al., 2019), and artificial light-harvesting systems (Xiao et al., 2019g, 2020a). Particularly, the development of fluorescent supramolecular polymers (FSPs) has attracted more and more interest because of their potential application in the area of dynamic luminescent materials (Ji et al., 2013; Zhang et al., 2017, 2018; Li et al., 2019). Moreover, the emergence of aggregation-induced emission (Hong et al., 2011) (AIE) fluorophores laid the foundation for the application of FSP in practice.

Macrocycle-based host-guest interaction is an important driving force in supramolecular chemistry. For example, we have reviewed a series of supramolecular materials based on pillararene (Xiao et al., 2019a,f,h). Benzo-21-crown-7 (B21C7) is one of the most important crown ethers (Zhang et al., 2007), and it shows interesting applications like adhesive materials (Dong et al., 2017; Zhang et al., 2019). B21C7 is the smallest crown ether that can complex with dialkylammonium salts, leading to a relatively strong host-guest interaction. Cyanostilbene is a well-known fluorophore and shows interesting AIE behavior, which is usually used for the construction of rotaxanes (Lee et al., 2013), nanoparticles with near-infrared emission (Shi et al., 2016), hydrogen-bonded supramolecular polymer (Lavrenova et al., 2017), and light-harvesting system (Kim et al., 2018; Sun et al., 2020). To the best of our knowledge, the integration of B21C7 unit and cyanostilbene unit to a host molecule to construct FSP has not yet been reported so far.

**Figure 1:**
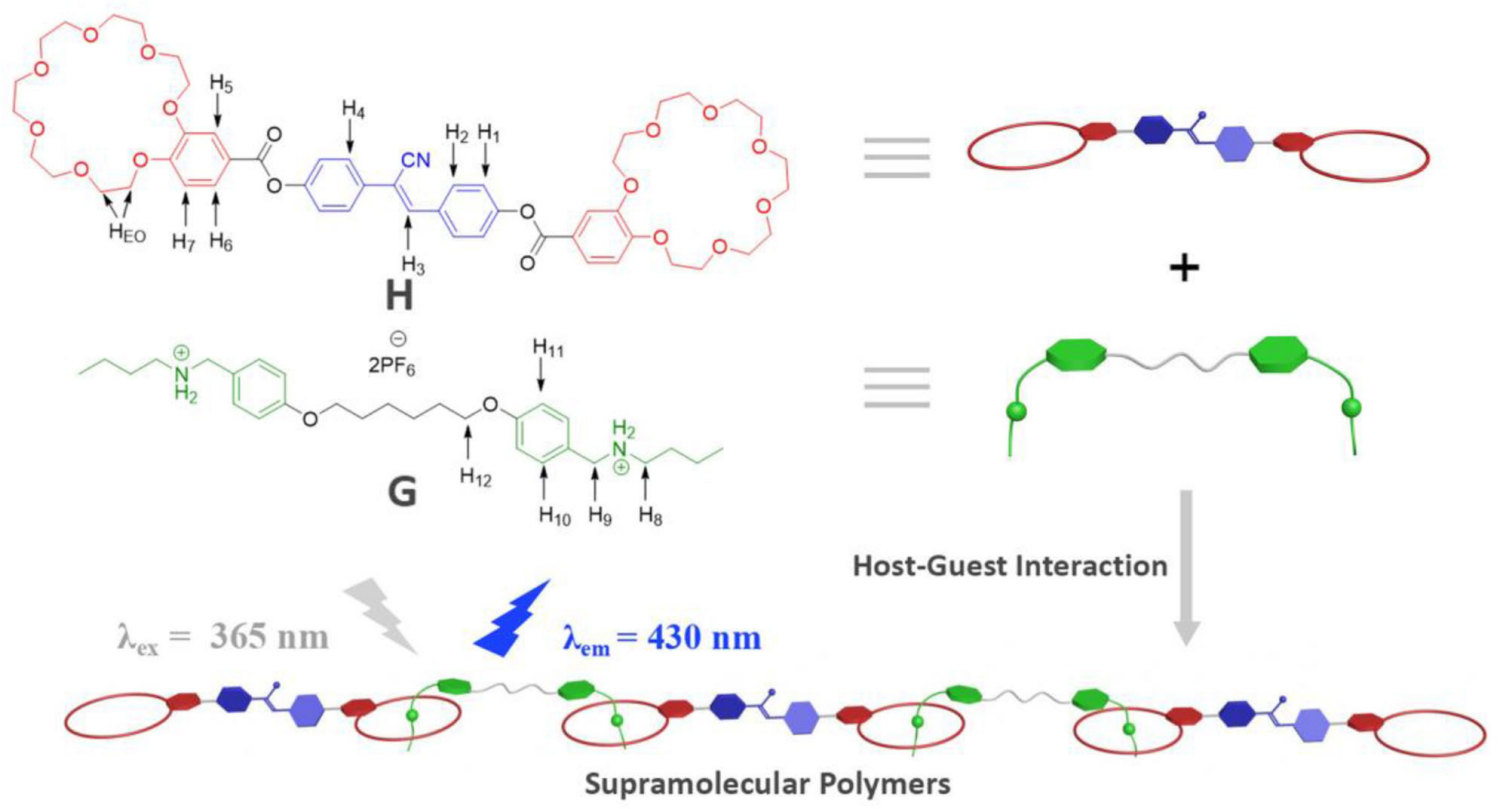
Schematic illustration of the AIE supramolecular polymer constructed from **H** and **G**.

Previously, we have reported some orthogonal supramolecular polymers based on B21C7 (Xiao et al., 2013, 2020c). Herein, we designed and synthesized a new host molecule **H**, which bears both cyanostilbene motif and B21C7 units (Figure 1). The cyanostilbene motif endows **H** with the property of AIE, while B21C7 unit endows **H** with the capability to bind dialkylammonium salt. In the presence of guest molecule **G** (a ditopic dialkylammonium salt compound), a novel AA/BB-type FSP can be fabricated by crown ether-based host-guest complexation. Furthermore, the FSP could be assembled into macroscopic fibers with blue fluorescence from concentrated solution. This crown ether-based FSP with beautiful blue fluorescence both in solution and in the solid state may have potential application in supramolecular luminescent materials.

## Results and Discussions

### Synthesis

**Scheme 1:**
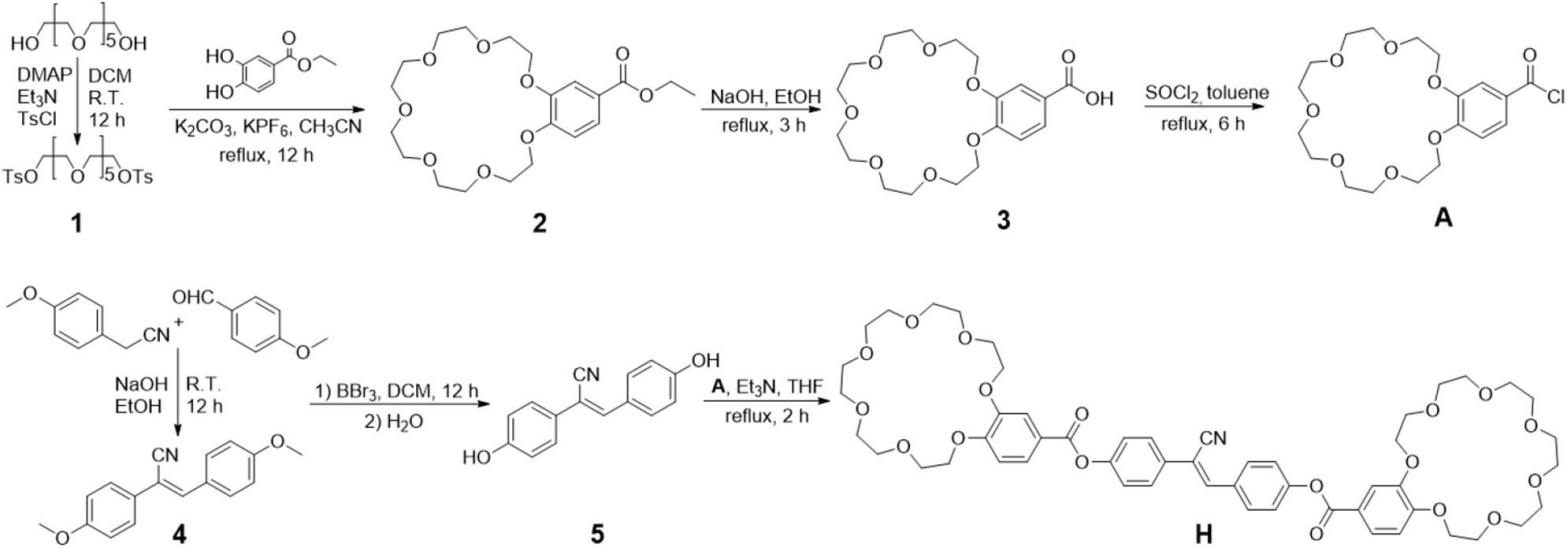
Synthetic route of compound **H**.

The synthesis of organic molecules **H** and **G** are straightforward. Compound **H** was synthesized from B21C7-based derivative **A** (Lu et al., 2018) and cyanostilbene-derived compound **5** (Scheme 1). As shown in Scheme 1, starting from p-anisaldehyde and 4-methoxybenzyl cyanide, compound **4** was prepared in ethanol solution by condensation reaction. Demethylation of compound **4** with boron tribromide in dichloromethane (DCM) yields compound **5** (Gu et al., 2012). Compound **G** was synthesized according to literature report (Li et al., 2018). The compounds that have not been reported previously are carefully characterized by ^1^H NMR, ^13^C NMR, and HR-MS (Supplementary Figures 1–4).

### AIE and Vapochromic Behavior of H

**Figure 2:**
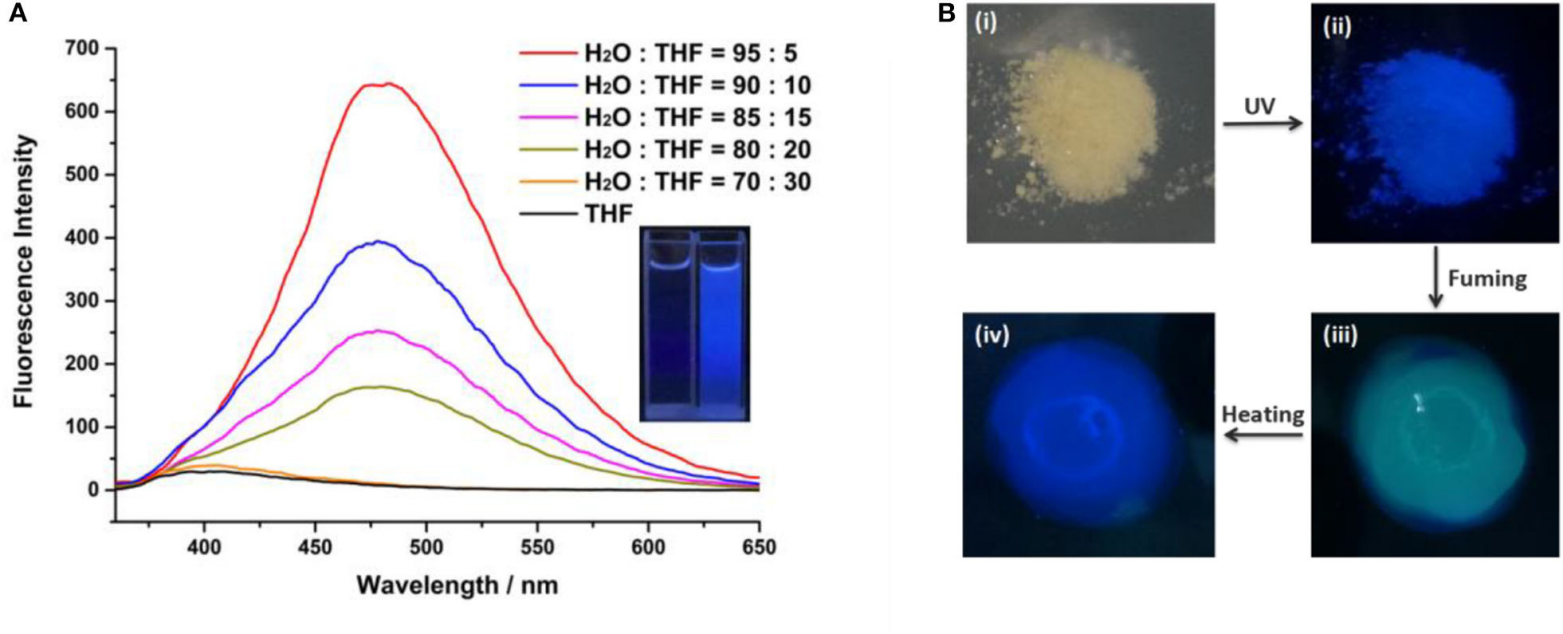
**(A)** Fluorescence emission spectra of **H** versus H_2_O fraction in THF/H_2_O mixtures (λ_ex_ = 365 nm, [**H**] = 5 × 10^−5^ M). Inset: photographs of **H** in THF and **H** in THF/H_2_O = 5: 95 mixtures ([**H**] = 5 × 10^−5^ M); and **(B)** solid powder of **H** under natural light (i) and UV light (ii), fumigation with DCM (iii), and color recovered by heating (iv).

In order to examine whether the cyanostilbene motif bridged B21C7 is AIE active, the fluorescence spectra of **H** in mixed H_2_O/Tetrahydrofuran (THF) solutions were investigated. As shown in Figure 2, **H** shows an obvious AIE behavior. There is no fluorescence emission when **H** was in pure THF (a good solvent for **H**). When water (a poor solvent for **H**) content was increased gradually to 80%, a moderate emission was observed. The absorption spectrum of **H** is shown in Supplementary Figure 5. The emission wavelength was at 480 nm when exited at 365 nm. Upon increasing water content to 95%, the fluorescence intensity of **H** exhibits a dramatic enhancement with a bright blue color. The dried powder of **H** was obtained as a light yellow solid, which exhibited intense blue luminescence under irradiation at 365 nm at room temperature (Figure 2B). Interestingly, exposing the sample to DCM vapor resulted in a distinct change of color from blue to green within only 30 s (Figure 2B). Notably, the blue color can be recovered by heating the sample to remove the DCM (Figure 2B).

### Supramolecular Polymerization Studied by ^1^H NMR and Viscometry

**Figure 3:**
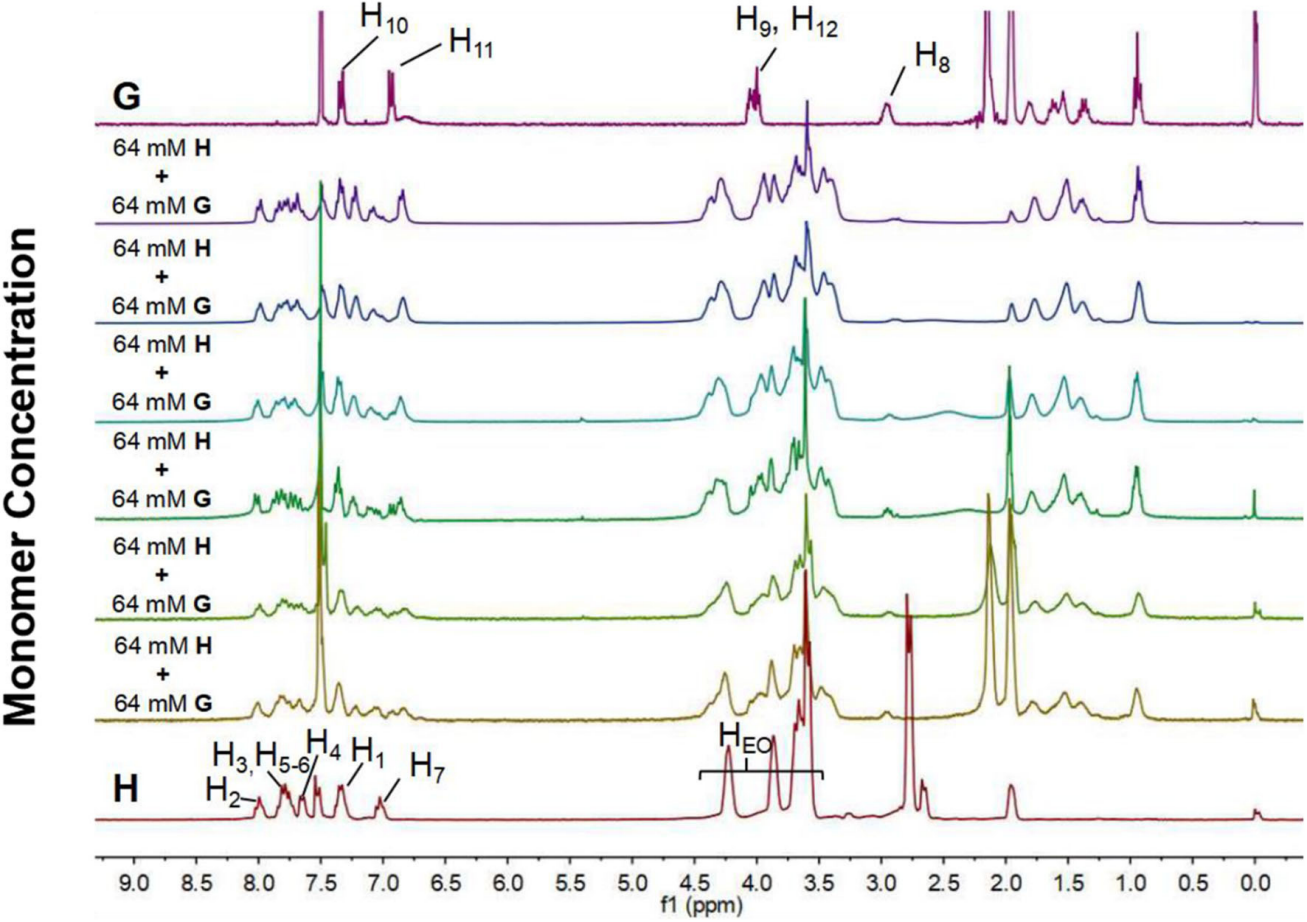
^1^H NMR spectra (300 MHz, CDCl_3_/CD_3_CN = 1/1, v/v, 298 K) of individual **H** and **G**, and mixtures of them at different monomer concentrations ([**H**]/[**G**] = 1/1).

Supramolecular polymerization of **H** and **G** was first investigated by concentration-dependent ^1^H NMR. It was measured in mixed CDCl_3_/CD_3_CN (1:1, v/v) at concentrations in the range of 2–64 mM (Figure 3). The concentration-dependent ^1^H NMR spectra showed a complex picture owing to the slow-exchange complexation of the B21C7 motif with the dialkylammonium salt on the NMR timescale. It should be noted that the peak splitting is relatively sharp at low concentrations (2–8 mM), suggesting that the cyclic oligomers are predominant species at this stage. As the concentration increases, these peaks became broad, indicating the formation of high-molecular-weight assemblies, such as supramolecular polymers. By contrast, the concentration-dependent ^1^H NMR spectra of individual **H** shows no chemical shift change upon concentration increasing (Supplementary Figure 6).

**Figure 4:**
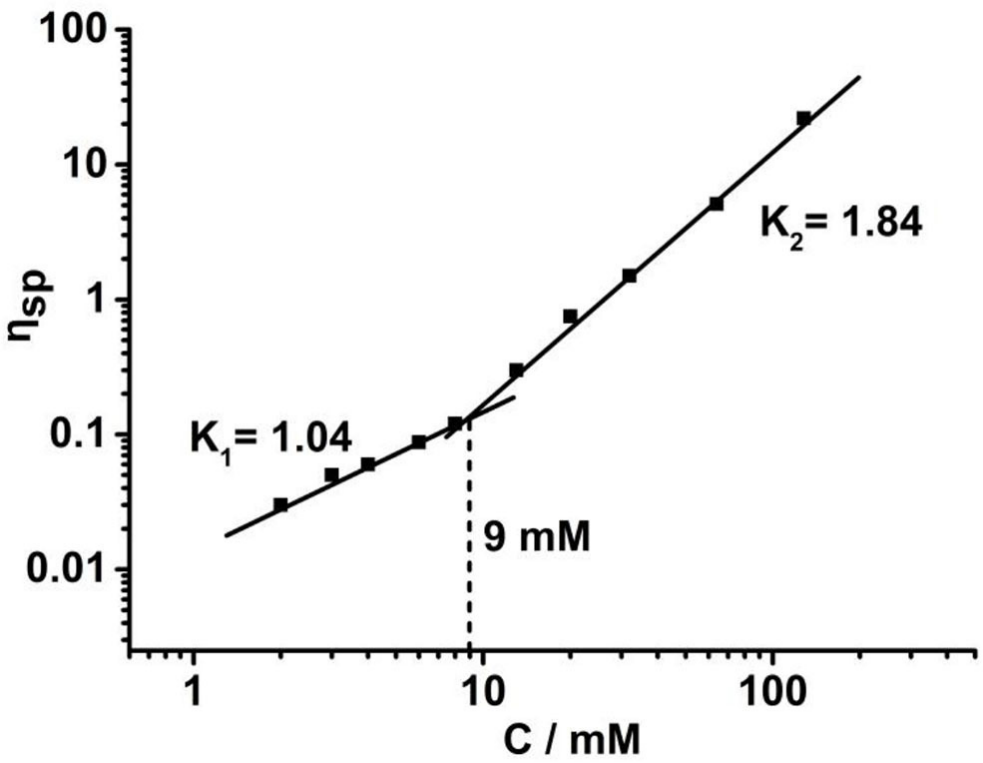
Specific viscosity of **H**/**G** complex vs. the monomer concentration in CDCl_3_/CH_3_CN (1:1, v/v) solutions (298 K).

To further study the supramolecular polymerization driven by crown ether-based host-guest interaction, viscosity measurements were performed by using a micro-Ubbelohde viscometer. A double logarithmic curve of specific viscosity toward monomer concentration is depicted in Figure 4. During the low concentration range, the slope value was tested to be 1.04, which is the characteristic of cyclic oligomers with constant size. When the concentration increased to 9 mM, a steeper curve with a slope 1.84 was obtained, suggesting that the cyclic oligomers are gradually transformed into supramolecular polymers. This phenomenon was in line with concentration-dependent ^1^H NMR.

### Solid-State Fluorescence Spectroscopy

**Figure 5:**
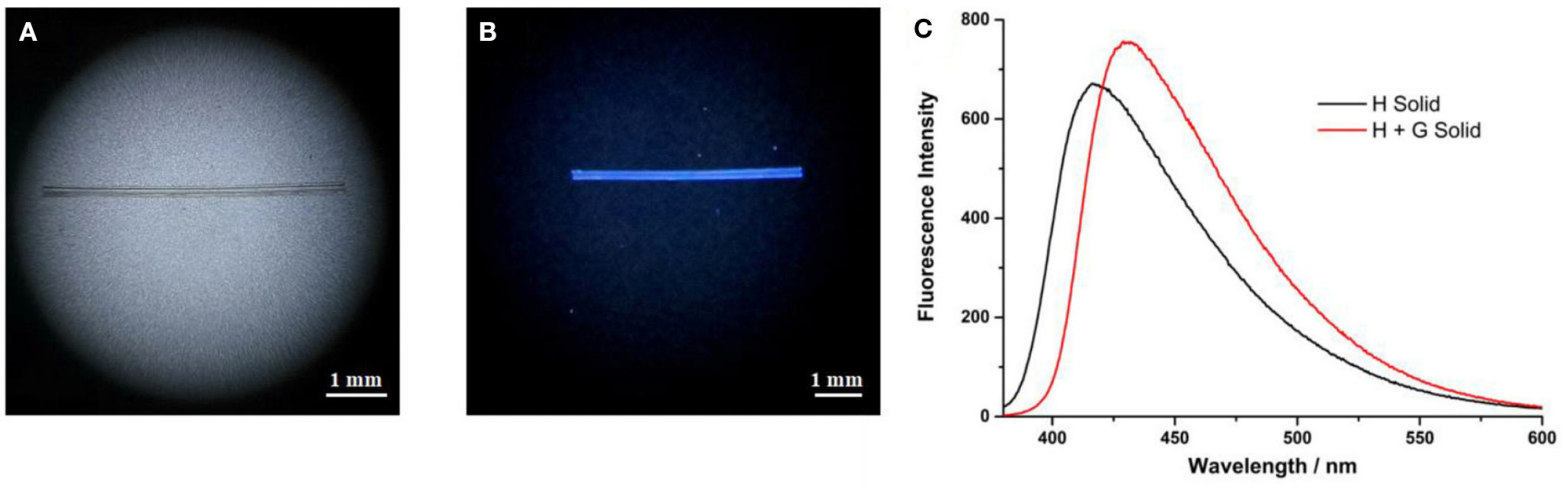
**(A)** A rod-like fiber formed from **H** and **G** under visible light; **(B)** the rod-like fiber under UV (365 nm) lamp irradiation; and **(C)** fluorescence spectra of **H** and **H-G** in the solid state, λ_ex_ = 365 nm.

The formation of supramolecular polymers was further evidenced by its processibility. Fibers can be drawn from a concentrated solution of the host and guest with a molar ratio of **H**/**G** = 1/1 (Figure 5A). Such fibers can be only made from entanglements of large aggregates. By contrast, no fiber can be pulled out from the concentrated solution of individual **H** or individual **G**. The fiber is colorless under visible light (Figure 5A) and generates blue fluorescence under UV light (Figure 5B). This fiber still has fluorescent luminescence after preparation for several days, indicating that the supramolecular polymer has potential applications in the area of supramolecular luminescent materials. The photophysical property of the supramolecular polymer was further investigated by solid-state fluorescence spectroscopy. As shown in Figure 5C, the emission wavelength of **H** shows a hypochromic shift from 480 nm in solution to 415 nm in the solid state. Compared with individual **H**, the supramolecular polymer in the solid state exhibited a stronger emission and bathochromic shift to 430 nm, indicating that the incorporation of the guest affected the packing of the fluorophores in the solid state, resulting in modified luminescent properties.

## Experimental

### General

All chemicals, reagents, and solvents were purchased from commercial suppliers and used, unless otherwise stated, without further purification. If needed, solvents were dried by literature-known procedures. All yields were given as isolated yields. The ^1^H NMR and ^13^C NMR spectra were recorded with a Bruker AVANCE III (300 MHz) spectrometer and calibrated against the residual proton signal or natural abundance carbon resonance of the used deuterated solvent from tetramethylsilane as the internal standard. The chemical shifts δ are indicated in ppm and the coupling constants *J* in Hz. The multiplicities are given as s (singlet), d (doublet), dd (doublet of doublets), t (triplet), and m (multiplet). High-resolution electrospray ionization mass spectra (HR-ESI-MS) were recorded on an Agilent Technologies 6540 UHD Accurate-Mass. Fluorescence measurements were performed on an Agilent Cary Eclipse spectrofluorometer. Viscosity measurements were carried out with Ubbelohde microviscometers (Shanghai Liangjing Glass Instrument Factory, 0.40 mm inner diameter) at 298 K in chloroform and acetonitrile.

### Synthesis of Compound H

To a solution of compound **5** (237 mg, 1.0 mmol) in THF (15 mL) was added compound **A** (1.26 g, 3.0 mmol) and 4-dimethylaminopyridine (18 mg, 0.15 mmol) at room temperature under N_2_ atmosphere. Then the Et_3_N (303 mg, 3.0 mmol) was added with vigorous stirred over 15 min. The reaction mixture was heated at 70°C for 2 h and then poured into water (100 mL). The resulting mixture was extracted with DCM (50 mL × 3) and the combined extracts were washed with H_2_O (100 mL × 3), brine (50 mL × 3), dried over anhydrous Na_2_SO_4_ and concentrated under reduced pressure. The resulting residue was chromatographed over silica gel (DCM:MeOH = 60:1, v/v) to afford compound **H** as a light yellow solid (460 mg, 0.46 mmol), yield: 46%. ^1^H NMR (300 MHz, CDCl_3_): δ (ppm) = 7.98 (d, *J* = 9.0 Hz, 2H, Ar*H*), 7.85 (dd, *J* = 8.4, 2.1 Hz, 2H, Ar*H*), 7.74 (d, *J* = 8.7 Hz, 2H, Ar*H*), 7.69 (d, *J* = 1.8 Hz, 2H, Ar*H*), 7.54 (s, 1H, alkene-*H*), 7.32 (m, 4H, Ar*H*), 6.96 (d, *J* = 8.7 Hz, 2H, Ar*H*), 4.33–4.19 (m, 8H, -OC*H*_2_C*H*_2_O-), 4.03–3.93 (m, 8H, -OC*H*_2_C*H*_2_O-), 3.82 (m, 8H, -OC*H*_2_C*H*_2_O-), 3.76 (m, 8H, -OC*H*_2_C*H*_2_O-), 3.69 (s, 16H, -OC*H*_2_C*H*_2_O-). ^13^C NMR (75 MHz, CDCl_3_): δ (ppm) = 164.6, 164.5, 153.7, 153.7, 152.6, 151.8, 148.5, 141.2, 132.0, 131.2, 130.6, 127.2, 124.9, 124.8, 122.6, 122.5, 121.7, 121.6, 117.9, 114.9, 112.3, 110.8, 71.4, 71.3, 71.2, 71.1, 71.0, 71.0, 70.6, 69.6, 69.5, 69.4, 69.2. HR-ESI-MS: *m/z* calcd for [C_53_H_64_NO_18_]^+^ = 1002.4118, found = 1002.4120.

## Conclusion

In summary, we have successfully synthesized a cyanostilbene motif bridged ditopic B21C7 compound, which exhibits good AIE property and vapochromic behavior. Supramolecular polymers can be fabricated by such a host compound with a ditopic dialkylammonium salt guest. The host-guest supramolecular polymerization was fully characterized by concentration-dependent ^1^H NMR, viscosity measurements, and fiber formation test. Fluorescent properties of the supramolecular polymers in the solid state are also studied, which suggests that the supramolecular polymers can enhance the AIE of the individual host. The FSP described in this work may have potential applications in the field of dynamic luminescent materials.

## Data Availability Statement

The original contributions generated for the study are included in the article/Supplementary Material, further inquiries can be directed to the corresponding author/s.

## Author Contributions

TX conceived and designed the study and wrote and revised the manuscript. HW conducted the synthetic experiments. All authors analyzed and interpreted the data.

## Conflict of Interest

The authors declare that the research was conducted in the absence of any commercial or financial relationships that could be construed as a potential conflict of interest.
