# Supplementary material for: Supramolecular Polymers With AIE Property Fabricated From a Cyanostilbene Motif-Derived Ditopic Benzo-21-Crown-7 and a Ditopic Dialkylammonium Salt
Source: Front Chem. 2020 Nov 19;8:610093. doi: 10.3389/fchem.2020.610093 (PMC7710702; doi:10.3389/fchem.2020.610093)
Supplement: Supplementary file 1 [file Table_1.DOCX]

**Supporting Information**

**Supramolecular Polymers with AIE property Fabricated from a Cyanostilbene Motif Derived Ditopic Benzo-21-Crown-7 and a Ditopic Dialkylammonium Salt**

Haoran Wu, Tangxin Xiao,*

*^a^School of Petrochemical Engineering, Changzhou University, Changzhou, 213164, China. E-mail: xiaotangxin@cczu.edu.cn*

**Abbreviations**

THF = Tetrahydrofuran; DCM = dichloromethane; M = mol/L; br = broad; Ar = aromatic group

DMAP = 4-Dimethylaminopyridine

## Synthesis of H


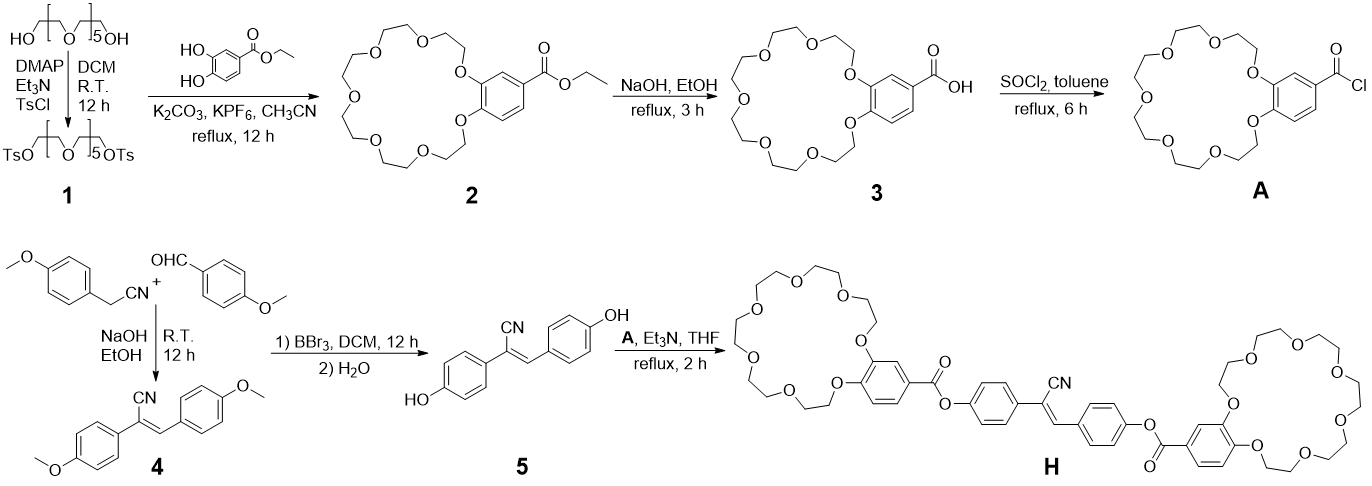


**Scheme S1**. Synthesis of **H**

Compound **A** was synthesized according to literature report^[S1]^

**Synthesis of compound 4**^[S2]^

To a flask equipped with a magnetic stirrer, p-anisaldehyde (4.59 g, 33.7 mmol), 4-methoxybenzyl cyanide (4.96 g, 33.7 mmol), NaOH (1.35 g, 33.7 mmol) and EtOH (100 mL) were charged under N_2_ atmosphere. The reaction was stirred overnight at room temperature. Filter to get product and wash with EtOH (3 × 10 mL), Vacuum drying to afford compound **4** as a light yellow solid (7.00 g, 26.4 mmol). Yield: 78%. ^1^H NMR (300 MHz, CDCl_3_): *δ* (ppm) = 7.85 (d, *J* = 8.7 Hz, 2H, Ar*H*), 7.58 (d, *J* = 9.0 Hz, 2H, Ar*H*), 7.36 (s, 1H, alkene-*H*), 6.98-6.94 (m, 4H, Ar*H*), 3.87 (s, 3H, OC*H_3_*), 3.85 (s, 3H, OC*H_3_*).


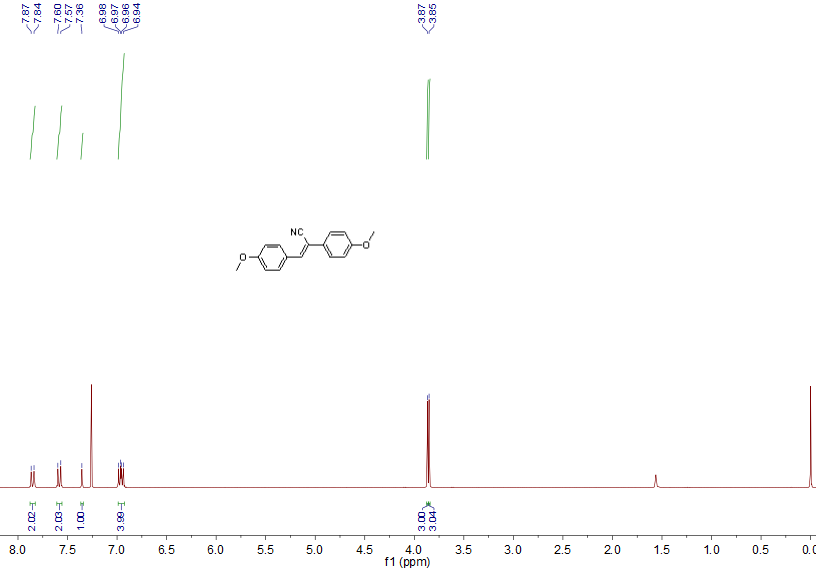


**Fig. S1.** ^1^H NMR spectrum (300 MHz, CDCl_3_, 298 K) of compound **4**.

**Synthesis of compound 5**^[S2]^

To a flask equipped with a magnetic stirrer, compound **4** (7.00 g, 26.4 mmol) and DCM (200 mL) were charged under N_2_ atmosphere. The mixture was cooled to 0 ℃ and then BBr_3_ (26.00 g, 105.5 mmol) was added with vigorous stirred over 30 min. After the system was warmed to room temperature, the reaction was stirred overnight. The flask was then immersed into an ice-water bath and water (50 mL) was dropwise added. The reacted mixture was extracted with DCM (3× 150 mL). The organic layer was isolated and washed with water three times. The organic layer was dried with anhydrous Na_2_SO_4_. With rotary evaporation, the crude product was obtained, which was purified by recrystallization with EtOH and water to afford compound **5** as a light yellow solid (4.50 g, 19.0 mmol). Yield: 72 %. ^1^H NMR (300 MHz, CDCl_3_): *δ* (ppm) = 10.12 (br, 1H, O*H*), 9.85 (br, 1H, O*H*), 7.79 (d, *J* = 9.0 Hz, 2H, Ar*H*), 7.67 (s, 1H, alkene-*H*), 7.53 (d, *J* = 8.7 Hz, 2H, Ar*H*), 6.90-6.84 (m, 4H, Ar*H*).

**
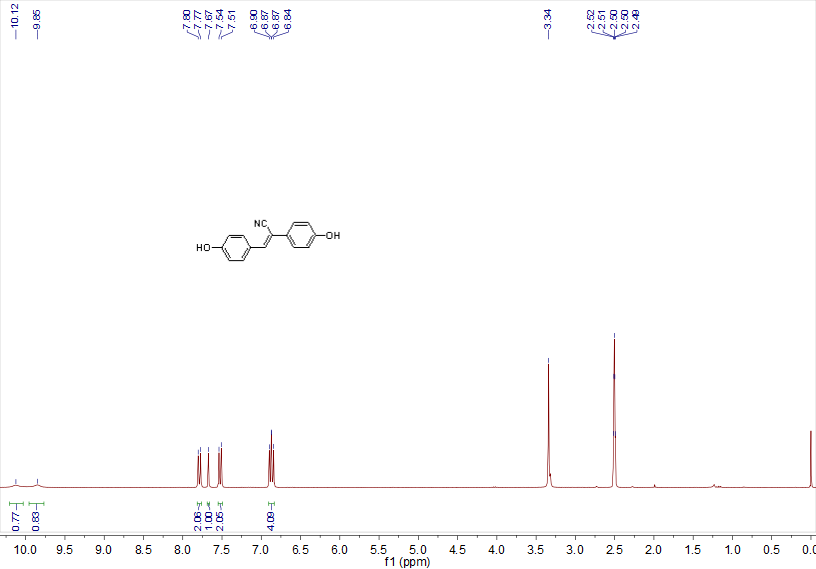
**

**Fig. S2.** ^1^H NMR spectrum (300 MHz, CDCl_3_, 298 K) of compound **5**.

**Synthesis of compound H**

To a solution of compound **5** (237 mg, 1.0 mmol) in THF (15 mL) was added compound A (1.26 g, 3.0 mmol) and DMAP (18 mg, 0.15 mmol) at room temperature under N_2_ atmosphere. Then the Et_3_N (303 mg, 3.0 mmol) was added with vigorous stirred over 15 min. The reaction mixture was heated at 70 °C for 2 h and then poured into water (100 mL). The resulting mixture was extracted with DCM (50 mL × 3) and the combined extracts were washed with H_2_O (100 mL × 3), brine (50 mL × 3), dried over anhydrous Na_2_SO_4_ and concentrated under reduced pressure. The resulting residue was chromatographed over silica gel (DCM : MeOH = 60 : 1, v/v) to afford compound **H** as a white solid (460 mg, 0.46 mmol), yield: 46%. ^1^H NMR (300 MHz, CDCl_3_): *δ* (ppm) = 7.98 (d, *J* = 9.0 Hz, 2H, Ar*H*), 7.85 (dd, *J* = 8.4, 2.1 Hz, 2H, Ar*H*), 7.74 (d, *J* = 8.7 Hz, 2H, Ar*H*), 7.69 (d, *J* = 1.8 Hz, 2H, Ar*H*), 7.54 (s, 1H, alkene-*H*), 7.32 (m, 4H, Ar*H*), 6.96 (d, *J* = 8.7 Hz, 2H, Ar*H*), 4.33 – 4.19 (m, 8H, -OC*H_2_*C*H_2_*O-), 4.03 – 3.93 (m, 8H, -OC*H_2_*C*H_2_*O-), 3.82 (m, 8H, -OC*H_2_*C*H_2_*O-), 3.76 (m, 8H, -OC*H_2_*C*H_2_*O-), 3.69 (s, 16H, -OC*H_2_*C*H_2_*O-). ^13^C NMR (75 MHz, CDCl_3_): *δ* (ppm) = 164.6, 164.5, 153.7, 153.7, 152.6, 151.8, 148.5, 141.2, 132.0, 131.2, 130.6, 127.2, 124.9, 124.8, 122.6, 122.5, 121.7, 121.6, 117.9, 114.9, 112.3, 110.8, 71.4, 71.3, 71.2, 71.1, 71.0, 71.0, 70.6, 69.6, 69.5, 69.4, 69.2. HR-ESI-MS: *m/z* calcd for [C_53_H_64_NO_18_]^+^ = 1002.4118, found = 1002.4120.


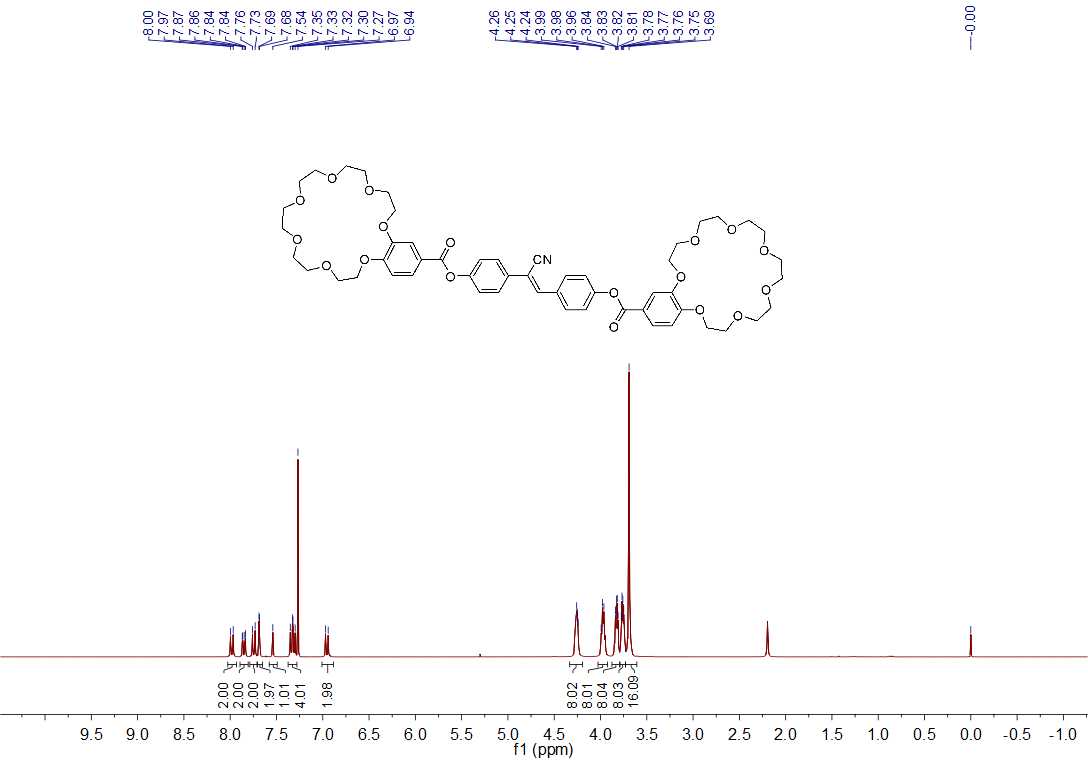


**Fig. S3.** ^1^H NMR spectrum (300 MHz, CDCl_3_, 298 K) of compound **H**.


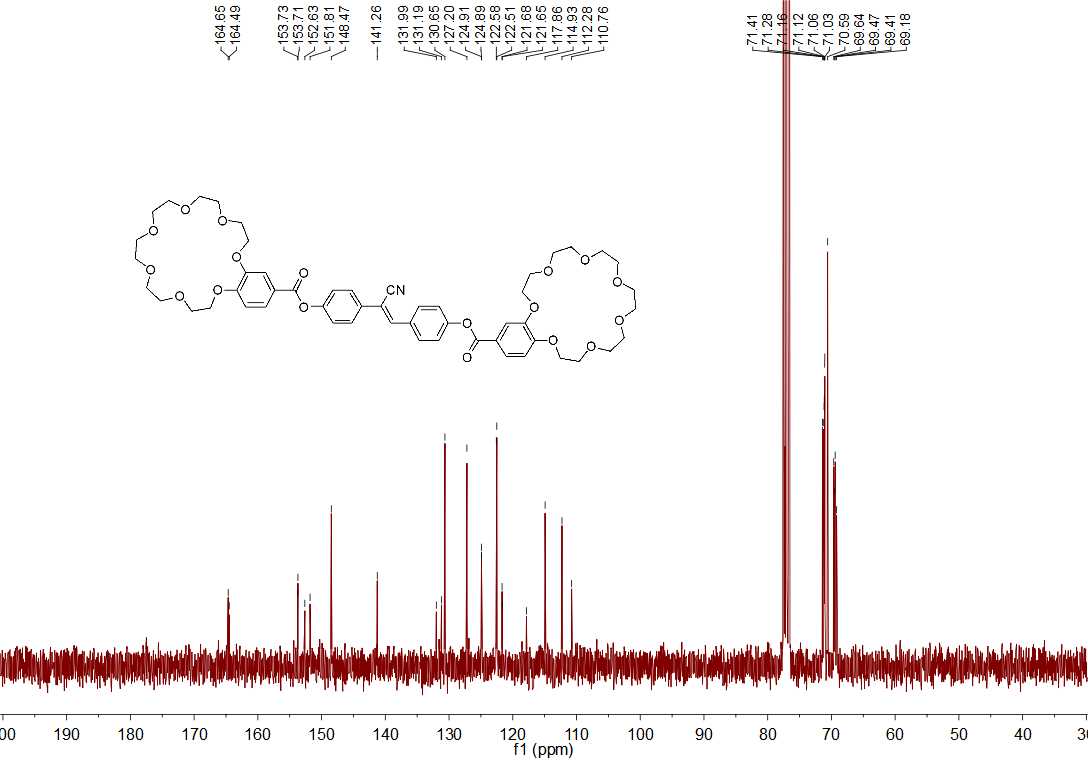


**Fig. S4.** ^13^C NMR spectrum (75 MHz, CDCl_3_, 298 K) of compound **H**.

## Absorption spectrum of H


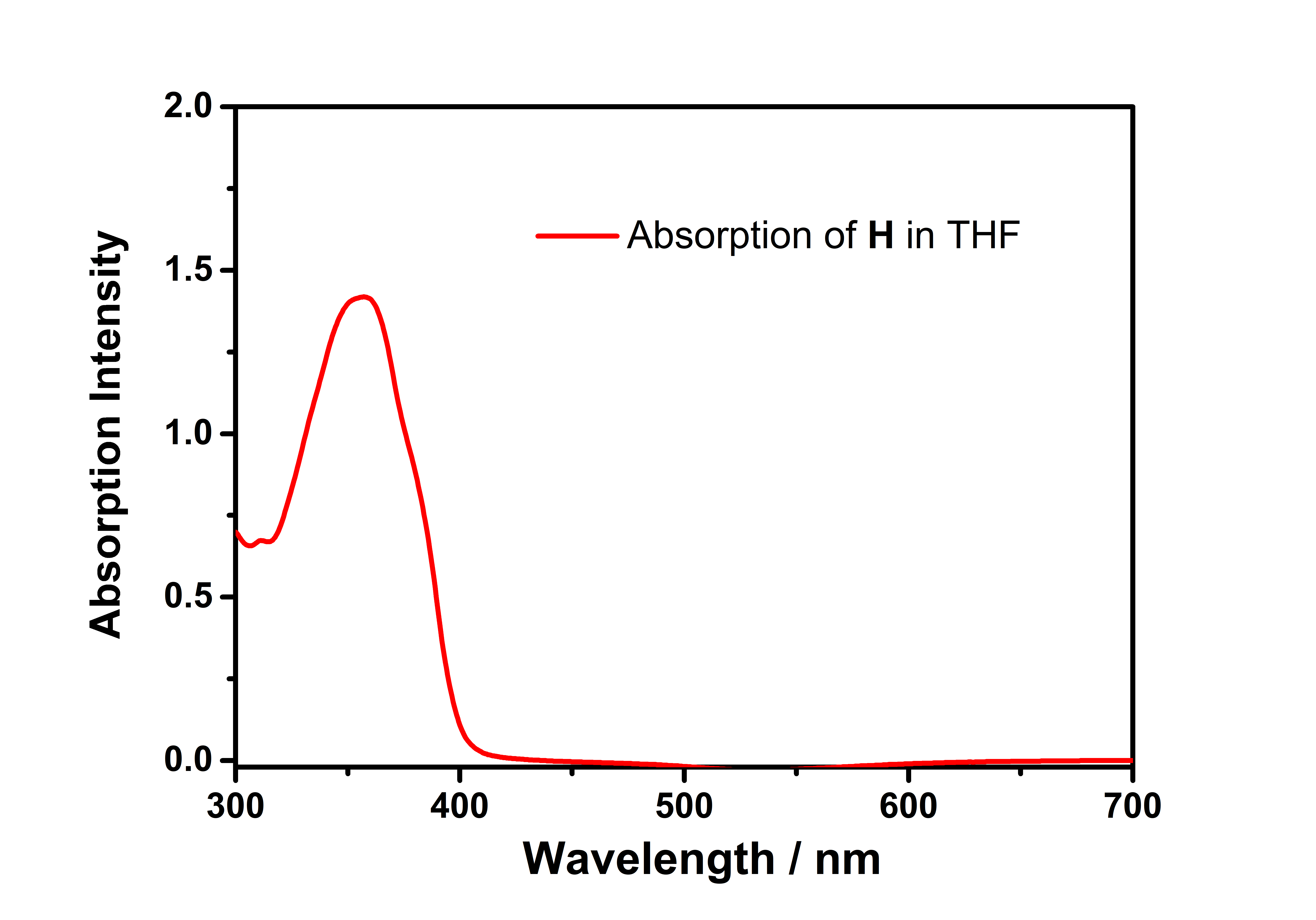


**Fig. S5.** Absorption spectrum of compound **H**.

## Concentration-dependent ^1^H NMR of H


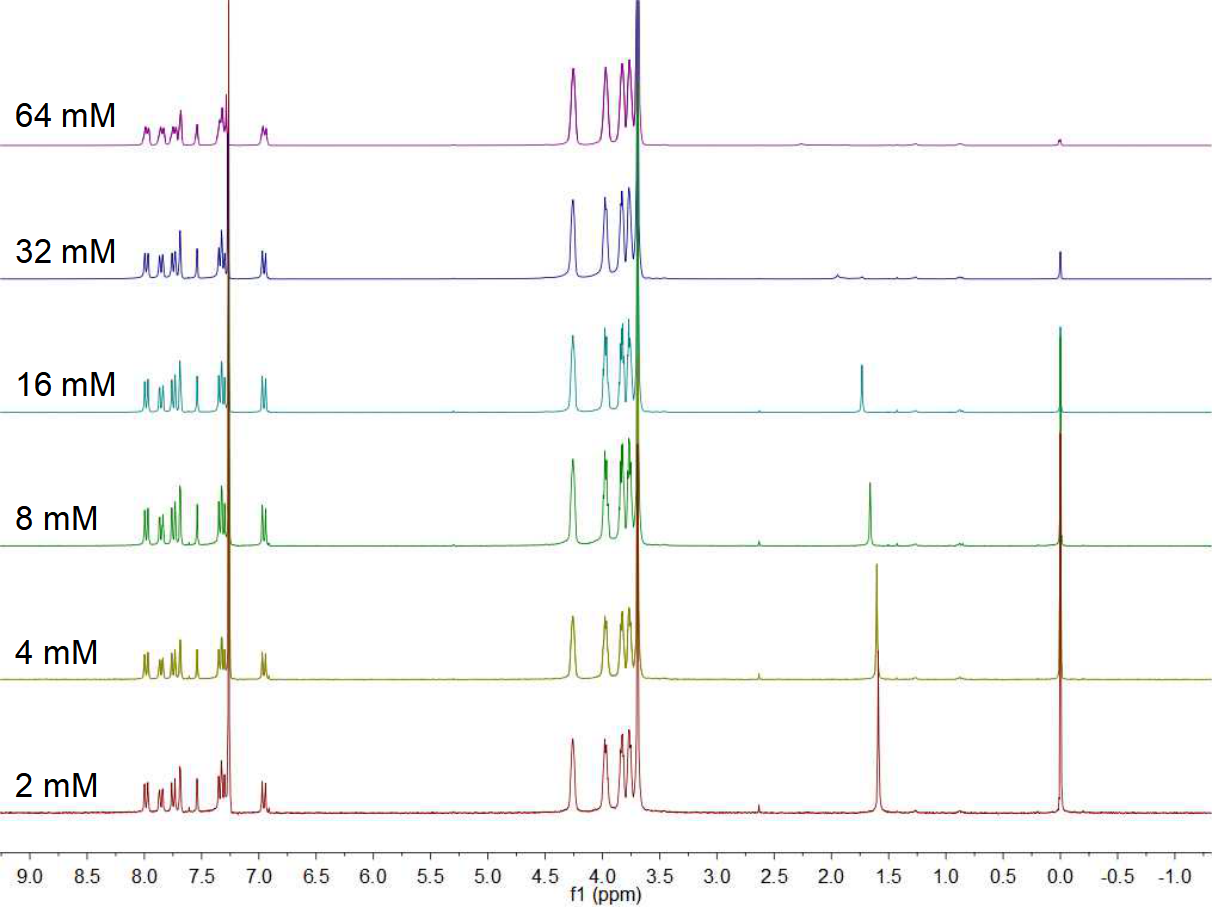


**Fig. S6.** ^1^H NMR spectra (300 MHz, CDCl_3_, 298 K) of individual **H** at different concentrations.

## References

[S1] Lu, C.; Zhang, M.; Tang, D.; Yan, X.; Zhang, Z.; Zhou, Z., et al., *J. Am. Chem. Soc.,* 2018, **140**, 7674-7680.

[S2] Meyers, Marvin J., et al., *J. Med. Chem.*, 2001, **44**, 4230-4251.
